# Dosimetry of inhaled ^**219**^Rn progeny

**DOI:** 10.1093/jrr/rraa140

**Published:** 2021-01-29

**Authors:** Hamadou Issa, Atangana Bingana Martin Serge, Saïdou

**Affiliations:** Nuclear Physics Laboratory. Faculty of Science. University of Yaoundé I. P.O. Box 812 Yaoundé. Cameroon; Nuclear Physics Laboratory. Faculty of Science. University of Yaoundé I. P.O. Box 812 Yaoundé. Cameroon; Nuclear Physics Laboratory. Faculty of Science. University of Yaoundé I. P.O. Box 812 Yaoundé. Cameroon; Nuclear Technology Section. Institute of Geological and Mining Research. P.O. Box 4110 Yaoundé. Cameroon

**Keywords:** actinon, ^211^Pb, ^211^Bi, inhalation, biokinetic models, dosimetric models, dose coefficient

## Abstract

During prostate cancer treatment with ^223^Ra. ^219^Rn (actinon) occurs and may be exhaled by the patient. Nurses and other hospital employees may inhale this radionuclide and its decay products. The alpha-emitting decay products of actinon deposited within a body will irradiate tissues and organs. Therefore. it is necessary to evaluate organ doses of actinon progeny. The purpose of this study is to set up a dosimetric method to assess dose coefficients for actinon progeny. The effective dose coefficients were calculated separately for three modes. The unattached mode which concerned the activity median thermodynamic diameter (AMTD) of 1 nm. and the nucleation and accumulation modes which are represented by activity median aerodynamic diameters (AMAD) of 60 and 500 nm respectively. The recent biokinetic models of actinon progeny developed in the Occupational Intakes of Radionuclides (OIR) publications series of the International Commission of Radiological Protection (ICRP) were implemented on BIOKMOD (Biokinetic Modeling) to calculate the number of nuclear transformations per activity intake of actinon progeny. The organ equivalent and effective dose coefficients were determined using the dosimetric approach of the ICRP. The inhalation dose coefficients of actinon progeny are dominated by the contribution of lung dose. The calculated dose coefficients of ^211^Pb and ^211^Bi are 5.78 × 10^−8^ and 4.84 × 10^−9^ Sv.Bq^−1^ for unattached particles (AMTD = 1 nm). and 1.4 × 10^−8^ and 3.55 × 10^−9^ Sv.Bq^−1^ for attached particles (AMAD = 60 nm). and 7.37 × 10^−9^ and 1.91 × 10^−9^ Sv.Bq^−1^ for attached particles (AMAD = 500 nm). These values are much closer to those of the recently published ICRP 137.

## INTRODUCTION

**Fig. 1.**
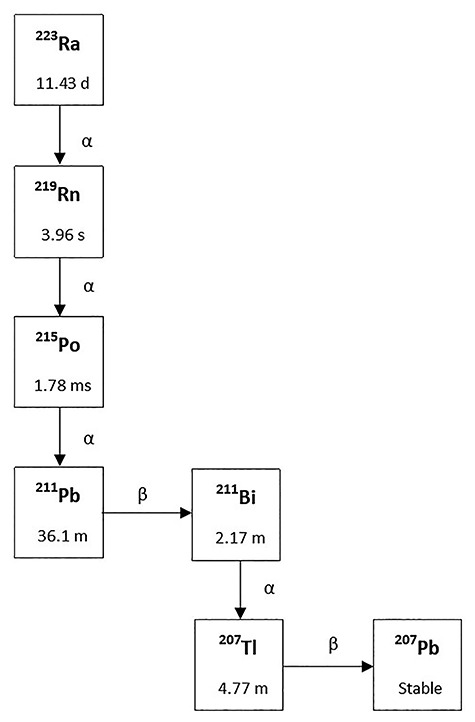
Decay chain of ^223^Ra with half-lives and decay-energy types [1, 2].

Actinon (^219^Rn) is a radioactive noble gas and a decay product of ^223^Ra in the ^235^U decay chain. ^219^Rn decays through the short-lived progeny ^215^Po. ^211^Pb. ^211^Bi and ^207^Tl to the stable nuclide ^207^Pb (Fig. 1). In contrast to radon (^222^Rn) and thoron (^220^Rn) which leave the soil and building materials and enter into the atmosphere. ^219^Rn due to its very short half-life (3.96 s) is generally less able to emanate from mineral matrices. Because of typically very low concentrations in the ambient air. exposure to ^219^Rn and its progeny are usually neglected. Thus. the measurement of ^219^Rn has not been described in standards such as ICRP 137 or ICRU 88 [1, 3]. Recently. in hospitals cancer treatment with ^223^Ra (Xofigo) was introduced [4, 5]. ^223^Ra is injected into patients to fight against bone metastasis of prostate cancer. In the decay chain. ^219^Rn occurs which may be exhaled by the patient. Secondary exposure of care-takers in the hospital and at home may happen by inhalation of actinon and its decay products.

Whereas many papers have been published on dosimetric studies of radon. thoron and their decay products. the International Commission on Radiological Protection (ICRP) has published inhalation dose coefficients of decay products of actinon (^211^Pb and ^211^Bi) using the size characteristics of radon progeny [1].

There are several pieces of software for internal dose assessment. However. most of them are commercialized. In the present work. the models for inhalation of actinon progeny have been mathematically implemented using a freely available package ICRP130Models on the recent version of BIOKMOD (version 5.4). and a dosimetric method is established to evaluate inhalation dose of actinon progeny using Microsoft excel. To make it effective. the effective doses and the organ equivalent doses in lung and in other organs of inhaled ^219^Rn progeny such as ^211^Pb and ^211^Bi were determined separately as a function of particle size distribution of three modes. using the human respiratory tract model (HRTM). the human alimentary tract model (HATM) and systemic models developed by ICRP [1, 6, 7]. The aim of this study is to determine the inhalation dose coefficients of actinon decay products. This approach could be used to also determine the dose conversion coefficient of actinon progeny for a real situation of secondary exposures of nurses at hospitals or the members of public at home.

## MATERIAL AND METHODS

In this section the internal dose calculations of actinon progeny are introduced. First the deposition fractions in the different regions of HRTM are presented. Then the biokinetic models describing dissolution. absorption and elimination of deposited material in the human body were implemented. and the activities occurring within the organs or tissues were calculated. Finally. the dosimetric model was applied to assess organ equivalent dose and effective dose coefficients with the calculation of radiation weighted *S* coefficient values. denoted *S*_w_. or specified source and target organs which were derived from the new ICRP voxel computational phantoms for a reference adult [8].

### Aerosol fractional deposition in human respiratory tract

Up to now there is no activity size measurement of actinon progeny. Due to its short half-life. ^219^Rn will probably not be able to escape from the point where it is formed. Therefore. ^219^Rn and its progeny are very rare in the ambient air. As the lead (^211^Pb) decay product of actinon has a half-life of 36.1 min. which is much closer to that of the lead (^214^Pb) progeny of radon (^222^Rn) with a half-life of 26.8 min. the activity size for radon progeny was used as the deposition fraction in the respiratory tract region. as displayed in Table 1 [1].

**Table 1:** Deposition fraction in the HRTM [1]. A breathing rate of 1.2 m^3^.h^−1^ is assumed. Geometric standard deviation (GSD) of 1.3 and 2.0 for unattached and attached particles. respectively. a unit of density and a shape factor are used for all modes.

Deposition fraction (%)			
Region^a^	AMTD (1 nm)	AMAD (60 nm)	AMAD (500 nm)
ET_1_	51.91	3.85	10.68
ET_2_	27.96	2.08	5.75
BB	7.93	0.93	0.60
bb	10.05	6.53	1.42
AI	0.59	27.90	9.05
Total	**98.43**	**41.29**	**27.51**

^a^ET_1_. anterior nasal passage; ET_2_. posterior nasal passage. pharynx and larynx; BB. bronchial; bb. bronchiolar. AI. alveolar interstitial.

### Biokinetic models of actinon progeny

**Fig. 2.**
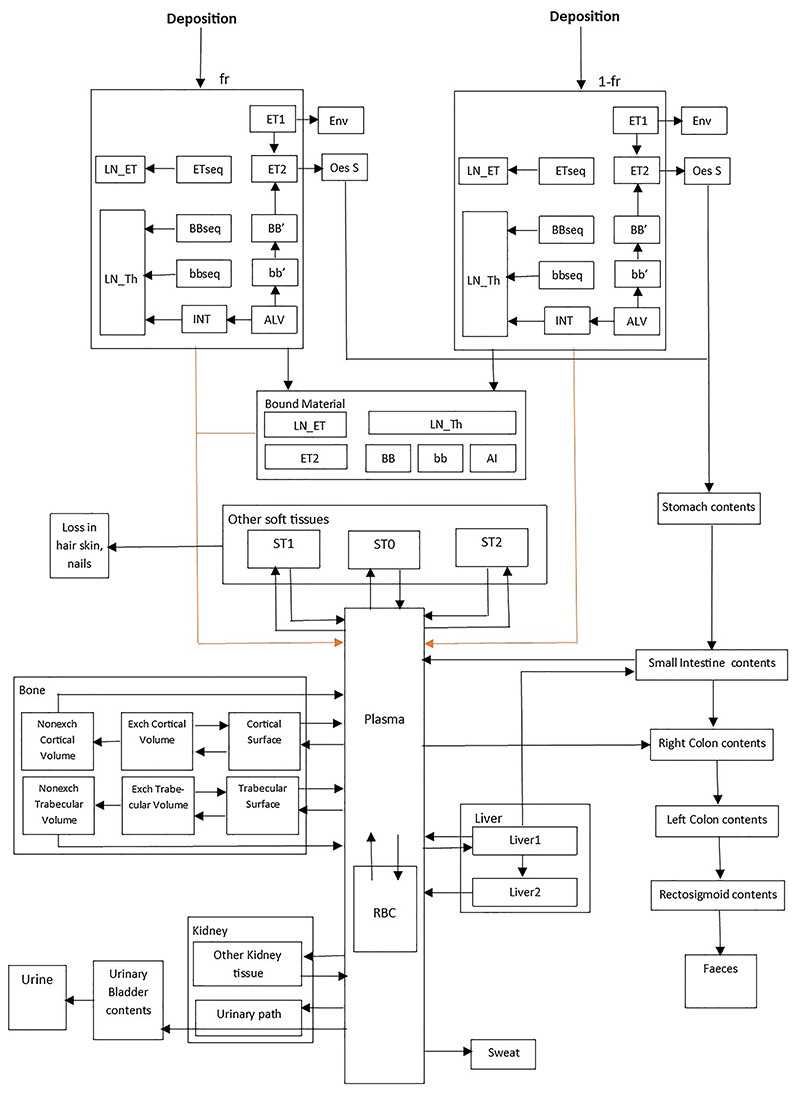
Inhalation biokinetic compartmental model for lead. It combines the HATM [9]. The systemic model of lead [1] and the HRTM [6, 7]. Extrathoracic region: ET_1_ = anterior nose), ET_2_ = posterior nasal passages, larynx, pharynx and mouth. ${\mathrm{LN}}_{\mathrm{ET}}$ = lymph nodes. Thoracic region: BB = bronchial, bb = bronchiolar, AI = alveolar-interstitial, ${\mathrm{LN}}_{\mathrm{TH}}$ = lymph nodes) [15]. ALV and INT = alveolar-interstitial. Other soft tissues: ST0 = soft tissue (fast turnover), ST1 = soft tissue (intermediate turnover), ST2 = soft tissue (slow turnover). Other compartments: Oes S = oesophagus slow, stomach contents, small intestine contents, right colon contents, left colon contents, rectosigmoid contents, faeces, sweat, urinary bladder contents, urine, loss in hair skin and nails, plasma. RBC = red blood cells. Bone: cortical surface. Exch Cortical Volume = exchangeable cortical volume, Nonexch Cortical Volume = Nonexchangeable cortical volume. Trabecular Surface. Exch Trabecular Volume = exchangeable trabecular volume, Nonexch Trabecular Volume = nonexchangeable trabecular volume. Liver: Liver1 and Liver2. Kidney: other kidney tissue, urinary path.

**Fig. 3.**
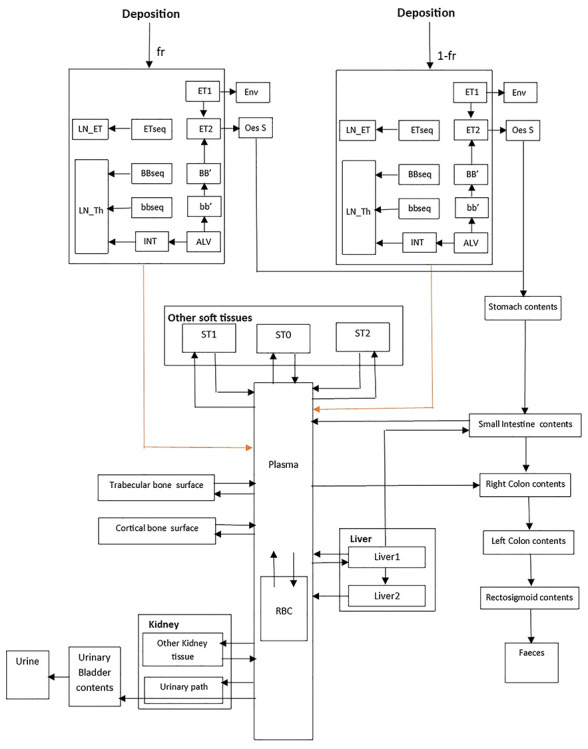
Inhalation biokinetic compartmental model for bismuth. It combines the HATM [9]. The systemic model of bismuth [1] and the HRTM [6, 7]. Extrathoracic region: ET_1_ = anterior nose, ET_2_ =posterior nasal passages, larynx, pharynx and mouth. ${\mathrm{LN}}_{\mathrm{ET}}=$ lymph nodes. Thoracic region: BB = bronchial, bb = bronchiolar, AI = alveolar-interstitial, ${\mathrm{LN}}_{\mathrm{TH}}$ = lymph nodes [15]. Other soft tissues: ST0 = soft tissue (fast turnover), ST1 = soft tissue (intermediate turnover), ST2 = soft tissue (slow turnover). Other compartments: Oes S = oesophagus slow. Stomach contents, small intestine contents, right colon contents, left colon contents, rectosigmoid contents, faeces, sweat, urinary bladder contents, urine, plasma. RBC = red blood cells. Bone: trabecular bone surface, cortical bone surface. Liver: Liver1 and Liver2. Kidney: other kidney tissue, urinary path.

The behavior of inhaled radioactive particles in the respiratory tract is described in the HRTM and some changes have been made in ICRP 130 [6, 7]. The systemic models for actinon progeny (^211^Pb and ^211^Bi) and the HATM are described respectively by ICRP 137 and ICRP 100 [1, 9]. The biokinetic models describing inhalation of each actinon progeny are represented in Fig. 2 for ^211^Pb and Fig. 3 for ^211^Bi. The dissolution and absorption parameter values of inhaled ^211^Pb were applied to ^211^Bi formed in the respiratory tract [7, 10]. The systemic model for bismuth as progeny of lead (bismuth formed within the body) is described in ICRP 137 [1].

The biokinetic model parameters. i.e. transfer rate. absorption parameter values of the actinon progeny between organs or tissues in the HRTM. HATM and systemic models were taken from ICRP publications [1, 7].

The dynamic behavior of decay products of actinon in the organism can be represented by a number of interconnected compartments with transfer coefficients describing the exchange of material. The transfer of inhaled actinon progeny between compartments can be modelled as systems of coupled. first-order differential equations. These systems have been implemented and solved in BIOKMOD; a computer tool developed by Sanchez using the Wolfram Mathematica programming language [11–13]. The general form of the rate of change of the radionuclide concentration *i*. can be written as in [14].

(1) \begin{equation*} \frac{dA_i(t)}{dt}={\sum}_r{k}_{r.i}{A}_r(t)-{\sum}_j{k}_{i.j}{A}_i(t)-\lambda{A}_i(t)+{b}_i(t) \end{equation*}

where ${A}_i$ is the retention in compartment *i*. ${k}_{i.j}$ is the transfer coefficient of material from compartment *i* to compartment *j* (the first term represents the inputs to the compartment *i* from the rest of compartments r$\ne$i and the second term represents the outputs from the compartment *i* to others compartments j$\ne$i). $\lambda$ is the physical decay constant and ${b}_i(t)$ is the input from outside. The initial conditions were determined by using the deposition fractions (Table 1). The number of nuclear transformations occurring in source region ${r}_S$ during the commitment period (18250 days for adults) denoted ${\overset{\sim }{A}}_i({r}_S)$ is given:

(2) \begin{equation*} \overset{\sim }{A}\left({r}_S\right)={\sum}_i{\int}_0^{18250}{A}_i(t) dt \end{equation*}

where 18 250 (50 years) represents the number of days which is the commitment period for an adult. The summation in the equation above is over the association of kinetic compartments *i* forming source regions r_s_. These numbers of nuclear transformations for actinon progeny were calculated by using Wolfram Mathematica software. The number of nuclear transformations per activity intake in the source region r_s_ is given in the following equation [8]:

(3) \begin{equation*} \overset{\sim }{a\ }\left({r}_S\right)=\frac{\overset{\sim }{A}\left({r}_S\right)}{\sum_i{A}_i(0)} \end{equation*}

where ${A}_i(0)$ represents the initial deposition fraction in each compartment *i*.

**Table 2:** Inhalation dose coefficients (Sv.Bq^−1^$)$of ^211^Pb and ^211^Bi as a function of particles size in unattached (1 nm) and attached (60 nm and 500 nm)

Organ	^211^Pb			^211^Bi		
	AMTD (1 nm)	AMAD (60 nm)	AMAD (500 nm)	AMTD (1 nm)	AMAD (60 nm)	AMAD (500 nm)
Remainder tissues^a^^,^^b^	1.64E-08	2.74E-09	9.35E-09	2.64E-09	4.70E-10	1.94E-09
Colon	4.41E-11	5.30E-10	3.35E-10	1.25E-13	2.64E-13	1.69E-13
Lungs	4.65E-07	1.12E-07	5.11E-08	3.76E-08	2.91E-08	1.39E-08
R-Marrow	2.98E-11	3.53E-10	2.23E-10	8.34E-14	1.76E-13	1.13E-13
Endost-BS	2.54E-11	3.19E-10	2.02E-10	4.09E-14	8.66E-14	5.56E-14
Brain	1.41E-11	1.74E-10	1.10E-10	3.80E-14	8.04E-14	5.16E-14
S-Glands	1.36E-11	1.68E-10	1.06E-10	3.65E-14	7.73E-13	4.96E-14
Thyroid	2.72E-11	3.24E-10	2.04E-10	7.62E-14	1.61E-13	1.03E-13
Breast	9.10E−12	1.11E-10	7.02E-11	2.52E-14	5.35E-14	3.43E-14
Liver	3.35E-11	3.98E-10	2.51E-10	9.89E-14	2.09E-13	1.34E-13
UB-Wall	1.18E-11	1.58E-10	9.98E-11	3.26E-14	6.90E-14	4.43E-14
Gonads	2.25.E-11	2.75E-10	1.73E-10	6.33E-14	1.34E-13	8.60E-14
Skin	1.59E-11	1.90E-10	1.20E-10	4.21E-14	8.91E-14	5.71E-14
Oesophagus	9.55E-11	4.48E-10	3.03E-10	1.41E-13	2.29E-13	1.69E-13
St-stem	1.53E-10	5.57E-10	3.89E-10	1.29E-13	2.68E-13	1.74E-13
ET airways	2.13E-07	2.89E-08	1.17E-07	3.44E-08	6.11E-09	2.52E-08
Kidneys	2.66E-10	3.53E-10	2.23E-09	1.32E −13	2.81E-13	1.80E-13
Adrenals	3.17E-11	3.87E-10	2.44E-10	9.23E-14	1.95E-13	1.25E-13
O mucosa	1.53E-11	1.74E-10	1.15E-10	3.82E-14	7.93E-13	5.13 E-14
SI-stem	5.86E-11	5.80E-10	3.70E-10	1.35E −13	2.85E-13	1.83E-13
Lymphatic N	1.63E-11	1.74E-10	1.10E-10	4.51E-14	9.91E-14	6.22E-14
Heart-wall	3.53E-11	3.56E-10	2.25E-10	8.01E-14	1.69E-13	1.08E-13
Thymus	1.76E-11	1.76E-10	1.11E-10	3.68E-14	7.80E-14	5.00E-14
Spleen	5.22E-11	1.70E-10	3.94E-10	1.52E-13	3.23E-13	2.07E-13
Pancreas	3.30E-11	3.97E-10	2.50E-10	9.49E-14	2.01E-13	1.28E-13
Prostate/uterus	9.20E-12	1.11E-11	1.00E-11	3.45E-14	7.32E-14	4.69E-14
GB-wall	1.39E-11	1.69E-10	1.07E-10	3.66E-14	7.76E-13	4.98E-14
Muscle	1.24E-11	1.51E-10	9.55E-11	3.20E-14	6.77E-14	4.35E-14
Effective dose	5.78E-08	1.41E-08	7.43E-09	4.84E-09	3.55E-09	1.91E-09

^a^Remainder tissues: adrenals. extrathoracic regions of the respiratory tract. gall bladder. heart. kidneys. lymphatic nodes. muscle. oral mucosa. pancreas. prostate (male). small intestine. spleen. thymus and uterus/cervix (female)

^b^Lymphatic N = lymphatic nodes. O mucosa = oral mucosa. R-marrow = red marrow. St-stem = stomach. SI-stem = small intestine. UB-wall = urinary bladder wall. GB-wall = gall bladder wall. ET airways = extra thoracic airways. Endost-BS = endosteal cells. S Glands = Salivary Glands.

### Dosimetric models

This section presents the method used to calculate the radiation-weighting *S* coefficient. committed equivalent doses in each organ/tissue within the body and effective dose after inhalation of actinon decay products.

#### Radiation weighted *S* coefficient

The radiation weighted *S* coefficient ${S}_w({r}_T\leftarrow{r}_s)$ represents the time-dependent equivalent dose rate in the target tissue r_T_ per unit activity present in source tissue r_S_. ${S}_w({r}_T\leftarrow{r}_s)$ was calculated for each radiation type emitted by the actinon progeny. The general form of the ${S}_w$ coefficient is given by [8].

(4) \begin{equation*} {S}_w\left({r}_T\leftarrow{r}_s\right)={\sum}_R{w}_R{\sum}_i{E}_{Ri}{Y}_{Ri}\phi \left({r}_T\leftarrow{r}_S.{E}_{Ri}\right) \end{equation*}

where ${w}_R$ is the radiation weighting factor for radiation type R. ${E}_{Ri}$is the energy of the ${i}^{th}$radiation of type R emitted in the nuclear transformations of the radionuclide in joules (J); ${Y}_{Ri}$ is the yield of the ${i}^{th}$ radiation of type R per nuclear transformations (Bq.s^−1^); $\phi ({r}_T\leftarrow{r}_S.{E}_{Ri})$ is the specific absorbed fraction denoted as SAF which is defined as the fraction of energy ${E}_{Ri}$ of radiation type R emitted within the source tissue/organ r_s_ that is absorbed per mass in the target tissue ${r}_T$ (kg^−1^). ^211^Pb decays to the nuclide ^211^Bi through beta particle emission. In this case the spectral data are used in the calculation of ${S}_w$ instead of mean energy value [8]. ${S}_w$ for beta radiation is given by:

(5) \begin{equation*} {S}_w\left({r}_T\leftarrow{r}_s\right)={w}_{\beta }{\int}_0^{E_{max}}P(E) dE\ \phi \left({r}_T\leftarrow{r}_S.E\right) \end{equation*}

where *P*(*E*) is proportional to the probability that the beta particle will be emitted with kinetic energy between *E* and *E* + d*E*. *E* represents the beta energy. *P*(*E*) and *E* are taken from DECDATA software [16]. The calculation of integral over the beta particle spectrum is made by numerical methods. However. ^211^Bi decays to the nuclide of ^207^Tl through alpha particle emission and to ^211^Po through beta emission. The form of ${S}_w({r}_T\leftarrow{r}_s)$ of alpha emission is given by:

(6) \begin{equation*} {S}_w\left({r}_T\leftarrow{r}_s\right)={w}_{\alpha }{E}_{\alpha }{Y}_{\alpha }\ \phi \left({r}_T\leftarrow{r}_S.{E}_{\alpha}\right) \end{equation*}

${w}_{\alpha .\beta .\gamma }$ values (radiation weighting factor) are taken form ICRP publication 103 [17]. The specific absorbed fractions values are taken from the electronic data of ICRP 133 [8]. The linear interpolation was done on Microsoft excel to find each corresponding value of $\phi ({r}_T\leftarrow{r}_S.{E}_{Ri})$ to ${E}_{Ri}$.For the compartment called other soft tissue in the biokinetic models. including several source regions ${r}_s$. the specific absorbed fraction $\phi ({r}_T\leftarrow{r}_S)$ was calculated as [8]:

(7) \begin{equation*} \phi \left({r}_T\leftarrow Other\right)=\frac{1}{M_{other}}{\sum}_{r_s}{M}_{r_S}\ \phi \left({r}_T\leftarrow{r}_S\right) \end{equation*}

#### Committed equivalent dose and committed effective dose

The committed equivalent dose h (${r}_T)$ in the target region was calculated for reference adult male ${h}^M({r}_T)$ and reference adult female ${h}^F({r}_T)$ as [8]:

(8) \begin{equation*} {h}^M\left({r}_T\right)={\sum}_{r_S}\overset{\sim }{a\ }\left({r}_S\right){S}_W^M\left({r}_T\leftarrow{r}_S\right) \end{equation*}

(9) \begin{equation*} {h}^F\left({r}_T\right)={\sum}_{r_S}\overset{\sim }{a\ }\left({r}_S\right)\ {S}_W^F\left({r}_T\leftarrow{r}_S\right) \end{equation*}

where ${S}_W^M$(${r}_T\leftarrow{r}_S)$ and ${S}_W^F$(${r}_T\leftarrow{r}_S)$ are the *S* coefficients for male and female respectively. There is an exception for target regions consisting of several target tissues: extrathoracic region. lung. colon and lymphatic nodes. For each target region in those tissues there is an associated fractional weighting factor. The committed equivalent dose for those particular regions was calculated as:

(10) \begin{equation*} {h}_T^M={\sum}_{r_T}f\left({r}_T.T\right){h}^M\left({r}_T\right) \end{equation*}

(11) \begin{equation*} {h}_T^F={\sum}_{r_T}f\left({r}_T.T\right){h}^F\left({r}_T\right) \end{equation*}

where $f({r}_T.T)$ is the fractional weight. the values are taken from ICRP 133 [8]. The committed effective dose coefficient was calculated as [17]:

(12) \begin{equation*} e(50)={\sum}_T{w}_T\left(\frac{h_T^M+{h}_T^F}{2}\right) \end{equation*}

where ${w}_T$ is the weighting factor for tissue T taken from the ICRP publication [17]. Microsoft Excel was used to calculate the radiation weighting *S* coefficient. the committed equivalent dose coefficient and the committed effective dose coefficient after inhalation of the actinon progeny.

**Table 3:** Inhalation dose coefficients (Sv Bq^−1^) calculated in the present work and comparison to the results of ICRP 137

Mode	Dose coefficients (Sv/Bq)					
	Present study		ICRP-137		Differences (%)	
	^211^Pb	^211^Bi	^211^Pb	^211^Bi	^211^Pb	^211^Bi
Unattached	5.78E-08	4.84E-09	6.6E-08	4.8E-09	−12	0.8
Nucleation	1.41E-08	3.55E-09	2.2E-08	1.5E-09	−36	136
Accumulation	7.43E-09	1.91E-09	7.4E-09	5.3E-10	0.4	260

## RESULTS AND DISCUSSION

The calculated committed equivalent dose coefficients (male and female) from ^211^Pb and ^211^Bi for each target organ/tissue are shown in the Appendix (Tables A1 and A2). In general. the equivalent dose coefficients in the lung and extrathoracic tissues were relatively larger than in other organs for the three particles sizes (radon and thoron progeny as well). For the progeny of ^211^Pb. the equivalent doses in bronchi basal cells. bronchiole basal cells and ET_1_ basal cells were the highest for each mode: unattached. nucleation and accumulation respectively. However. for the progeny of ^211^Bi the ET_2_ basal cells. bronchiole basal cells and ET_2_ basal cells were the target regions where the equivalent dose coefficient was the highest for unattached (1 nm). nucleation (60 nm) and accumulation (500 nm) modes. respectively.

The committed equivalent dose in each tissue/organ for actinon progeny in unattached and attached modes are given in Table 2. For both actinon progeny the lung equivalent dose was the highest for unattached and nucleation modes. However. the ET airways (extrathoracic region) equivalent dose was the highest for accumulation mode. The lung dose strongly depends on the percentage of the deposition fraction within the bronchial and bronchiolar tissues. The organs and effective doses (dose coefficients) after inhalation actinon (^219^Rn) progeny. ^211^Pb and ^211^Bi in unattached and attached (nucleation and accumulation) modes are given in Table 2. The dose coefficient of short-lived actinon progeny was the highest for unattached mode (1 nm).

In this study. the lung equivalent dose for ^211^Pb (4.65 × 10^−7^ Sv.Bq^−1^) of the unattached mode was 4–9 times larger than the values (1.12 × 10^−7^ and 5.06 × 10^−8^ Sv.Bq^−1^) of nucleation and accumulation. For ^211^Bi. the lung equivalent dose (3.76 × 10^−8^ Sv.Bq^−1^) of the unattached mode was 1–3 times larger than those (2.91 × 10^−8^ and 1.39 × 10^−8^ Sv.Bq^−1^) of the other modes. For actinon progeny. organs such as kidneys. colon. stomach and oesophagus received relatively high doses compared to other organs. The inhalation dose coefficients of ^211^Pb and ^211^Bi for adults are 5.78 × 10^−8^ and 4.84 × 10^−9^ Sv.Bq^−1^ for unattached particles (1 nm). and 1.41 × 10^−8^ and 3.55 × 10^−9^ Sv.Bq^−1^ for attached particles (nucleation mode 60 nm) and 7.43 × 10^−9^ and 1.91 × 10^−9^ Sv.Bq^−1^ for attached particles (accumulation mode 500 nm). Those effective dose coefficients are in comparison with the values of 6.6 × 10^−8^ and 4.8 × 10^−9^ Sv.Bq^−1^ in the unattached mode (1 nm). 2.2 × 10^−8^ and 1.5 × 10^−9^ Sv.Bq^−1^ in the nucleation mode (60 nm) and 7.4 × 10^−9^ and 5.3 × 10^−10^ Sv.Bq^−1^ in the accumulation mode (500 nm) for ^211^Pb and ^211^Bi. respectively. calculated by ICRP 137 [1]. In Table 3 effective dose coefficients of actinon progeny calculated in this study were compared to those of ICRP 137 and the differences were found to be in the range −36 to 0.4% for ^211^Pb and 0.8–260% for ^211^Bi. The calculations were made using assumptions of the Occupational Intakes of Radionuclides (OIR) publications series. The differences found between the dose coefficients would come from the calculation pattern (the numerical integration of equation (5). the calculation of specific absorbed fractions $\phi ({r}_T\leftarrow Other)$ for the compartments denoted other soft tissues and the linear interpolation of specific absorbed fractions) and some details about the treatment of decay products formed in the respiratory tract. Apart of ^211^Bi. all other progeny radionuclides formed in the respiratory tract (^207^Tl and ^211^Po) after inhalation of ^211^Pb were neglected. In addition. the bound parameter values (transfer rate from the bound-state compartments to the body fluids as shown in Fig. 2) were also neglected for bismuth formed in the respiratory tract. Inhalation dose coefficients for actinon progeny were also calculated by Stabin and Siegel [19]. The dose coefficients were 3.46 × 10^−11^ and 1.76 × 10^−9^ Sv.Bq^−1^ for ^211^Pb and ^211^Bi. respectively [18]. One should note that Stabin and Siegel assessed the dose coefficients for one particles size mode (AMAD 5 μm) with the aerosol type ‘M’ in a particular situation of exposure. The dose coefficients (inhalation) of actinon progeny of tissues other than the lungs are ~5% of the total effective dose in the unattached and nucleation modes. while for the accumulation mode the coefficient is ~15% of the total effective dose. Overall the effective dose of inhaled actinon progeny was dominated by the lung equivalent dose. The extra-thoracic equivalent dose was of the same order of magnititude or lower than that to the lungs. However its contribution to the effective dose was quite low, because it is one of the 13 remainder organs (equivalent dose of remainder tissues is the arithmetic mean of the 13 equivalent doses of the remainder tissues) [17].

## CONCLUSION

This work presents internal dose calculations of inhaled actinon progeny. The effective dose coefficients were calculated separately for three modes. using biokinetic and dosimetric models developed in the recent OIR publications series of ICRP. The biokinetic models of ^211^Pb and ^211^Bi have been implemented and solved in BIOKMOD using the approach developed by Sanchez is described in references [11, 12]. The inhalation actinon progeny provided the highest dose to the lungs and ET airways. The calculations indicated that the most exposed region of the lung tissues for ^211^Pb was the bronchial tissue for the unattached and attached fractions respectively for particle sizes of 1 nm and 500 nm and the bronchiolar tissue for the attached fraction of 60 nm. However. the most exposed region of the lung tissues for ^211^Bi was the bronchiolar tissue for unattached fraction (1 nm) and bronchioles for attached fractions (60 nm and 500 nm particles size). The inhalation dose coefficients of actinon progeny found in this work were much closer to those of ICRP 137. Furthermore. in order to work out the dose conversion coefficient for actinon (^219^Rn) decay products. a study of activity size distributions and measurement of activity concentration of actinon progeny is recommended to be conducted in hospitals during the treatment of prostate cancer metastasis with ^223^Ra.

## CONFLICT OF INTEREST

None declared.

## Appendix

**Table A1:** Committed equivalent dose coefficients (Sv.Bq^−1^) for target region after inhalation ^211^Pb as decay product of actinon (reference adult male and female). *f*(r_T,_T)^M^h(r_T_) and *f*(r_T,_T)^F^h(r_T_) are committed equivalent dose coefficients for adult male and female respectively

Target tissue^a^	Unattached		Nucleation		Accumulation	
	*f*(r_T_.T)h^M^(r_T_)	*f*(r_T_.T)^F^h(r_T_)	f(r_T_.T)h^M^(r_T_)	*f*(r_T_.T)^F^h(r_T_)	*f*(r_T_.T)h^M^(r_T_)	*f*(r_T_.T)^F^h(r_T_)
O-mucosa	1.45E-11	1.62E-11	1.67E-10	1.82E-10	1.10E-10	1.21E-10
Oesophagus	1.16E-10	7.56E-11	4.15E-10	4.81E-10	2.91E-10	3.15E-10
St-stem	1.46E-10	1.61E-10	5.15E-10	6.01E-10	3.61E-10	4.18E-10
SI-stem	4.79E-11	6.95E-11	4.58E-10	7.04E-10	2.93E-10	4.48E-10
RC-stem	1.95E-11	1.68E-11	2.32E-10	1.97E-10	1.46E-10	1.24E-10
LC-stem	1.66E-11	1.88E-11	2.02E-10	2.30E-10	1.27E-10	1.45E-10
RS-stem	7.07E-12	9.41E-12	8.61E-11	1.15E-10	5.43E-11	7.24E-11
ET1-bas	8.19E-08	9.47E-08	1.45E-08	1.68E-08	6.03E-08	6.98E-08
ET2-bas	1.16E-07	1.34E-07	1.23E-08	1.43E-08	4.84E-08	5.61E-08
LN-ET	2.02E-12	1.14E-12	8.38E-12	8.94E-12	1.07E-11	7.23E-12
Bronch-bas	4.85E-08	5.40E-08	5.89E-09	6.61E-09	4.45E-09	4.99E-09
Bronch-sec	2.02E-07	2.24E-07	2.25E-08	2.51E-08	1.72E-08	1.92E-08
Bchiol-sec	1.97E-07	2.05E-07	7.67E-08	7.98E-08	2.53E-08	2.64E-08
AI	2.86E-10	3.61E-10	3.95E-09	4.86E-09	2.09E-09	2.58E-09
LN-Th	3.19E-12	1.20E-11	8.92E-12	1.60E-11	5.80E-12	1.10E-11
R-marrow	2.60E-11	3.36E-11	3.13E-10	3.94E-10	1.97E-10	2.49E-10
Endost-BS	2.21E-11	2.88E-11	2.79E-10	3.61E-10	1.76E-10	2.28E-10
Brain	1.31E-11	1.53E-11	1.61E-10	1.88E-10	1.01E-10	1.19E-10
Eye-lens	8.57E-12	9.85E-12	1.02E-10	1.22E-10	6.47E-11	7.67E-11
P-gland	8.44E-12	1.43E-11	7.00E-11	1.76E-10	5.56E-11	1.11E-10
Tongue	2.13E-11	1.50E-11	2.57E-10	1.77E-10	1.64E-10	1.14E-10
Tonsils	5.62E-12	1.45E-11	6.68E-11	1.76E-10	4.27E-11	1.12E-10
S-glands	1.31E-11	1.43E-11	1.61E-10	1.76E-10	1.02E-10	1.11E-10
Thyroid	2.44E-11	3.01E-11	2.94E-10	3.54E-10	1.85E-10	2.24E-10
Breast	1.31E-11	5.09E-12	1.61E-10	6.15E-11	1.02E-10	3.88E-11
Thymus	1.61E-11	1.91E-11	1.67E-10	1.85E-10	1.06E-10	1.18E-10
Ht-wall	2.79E-11	4.27E-11	3.05E-10	4.07E-10	1.93E-10	2.58E-10
Adrenals	2.95E-11	3.40E-11	3.60E-10	4.15E-10	2.27E-10	2.61E-10
Liver	2.89E-11	3.81E-11	3.45E-10	4.52E-10	2.18E-10	2.85E-10
Pancreas	2.99E-11	3.62E-11	3.60E-10	4.35E-10	2.27E-10	2.74E-10
Kidneys	2.45E-10	2.89E-10	3.24E-09	3.83E-09	2.05E-09	2.42E-09
Spleen	4.70E-11	5.75E-11	5.62E-10	6.88E-10	3.55E-10	4.34E-10
GB-wall	1.33E-11	1.45E-11	1.62E-10	1.78E-10	1.03E-10	1.12E-10
Ureters	5.62E-12	1.43E-11	6.66E-11	1.76E-10	4.21E-11	1.11E-10
UB-wall	1.07E-11	1.30E-11	1.43E-10	1.73E-10	9.02E-11	1.09E-10
Ovaries	0.00E+00	3.02E-11	0.00E+00	3.68E-10	0.00E+00	2.32E-10
Testes	1.48E-11	0.00E+00	1.82E-10	0.00E+00	1.15E-10	0.00E+00
Prostate	1.31E-11	0.00E+00	1.61E-10	0.00E+00	1.02E-10	0.00E+00
Uterus	0.00E+00	5.31E-12	0.00E+00	6.15E-11	5.97E-11	3.89E-11
LN-Sys	1.47E-11	1.80E-11	1.57E-10	1.92E-10	9.96E-11	1.21E-10
Skin	1.43E-11	1.77E-11	1.67E-10	2.15E-10	1.06E-10	1.36E-10
Adipose	1.14E-11	1.43E-11	1.24E-10	1.52E-10	7.91E-11	9.69E-11
Muscle	1.12E-11	1.37E-11	1.37E-10	1.66E-10	8.64E-11	1.05E-10

^a^O-mucosa = oral mucosa, St-stem = stomach, RC-stem = right colon, RC-stem = rectosigmoid colon, ET2-bas = ET2 basal cells, Bronch-bas = bronchi basal cells, Bchiol-sec = bronchiolar secretory cells, LN-Sys = lymph nodes thoracic, Endost-BS = endosteal cells, eye-lens = lens of eye, S-glands = salivary glands, Ht-wall = heart wall, GB-wall = gall bladder, UB-wall = urinary bladder.

**Table A2:** Committed equivalent dose coefficients (Sv.Bq^−1^) for target region after inhalation ^211^Bi as decay product of actinon (reference adult male and female). *f*(r_T,_T)h^M^(r_T_) and *f*(r_T,_T)h^F^(r_T_) are committed equivalent dose coefficients for adult Male and Female respectively.

Target Tissue	Unattached		Nucleation		Accumulation	
	*f*(r_T,_T)h^F^(r_T_)	*f*(r_T,_T)^M^h(r_T_)	*f*(r_T,_T)^F^h(r_T_)	*f*(r_T,_T)^M^h(r_T_)	*f*(r_T,_T)^F^h(r_T_)	*f*(r_T,_T)^M^h(r_T_)
O-mucosa	3.96E-14	3.68E-14	8.19E-14	7.66E-14	5.31E-14	4.96E-14
Oesophagus	1.51E-13	1.32E-13	2.46E-13	2.13E-13	1.81E-13	1.58E-13
St-stem	1.39E-13	1.20E-13	2.89E-13	2.49E-13	1.87E-13	1.61E-13
SI-stem	1.61E-13	1.09E-13	3.40E-13	2.32E-13	2.18E-13	1.49E-13
RC-stem	4.74E-14	5.36E-14	1.00E-13	1.13E-13	6.43E-14	7.27E-14
LC-stem	5.38E-14	4.77E-14	1.14E-13	1.01E-13	7.31E-14	6.48E-14
RS-stem	2.69E-14	2.09E-14	5.70E-14	4.43E-14	3.65E-14	2.84E-14
ET1-bas	6.02E-09	5.20E-09	1.06E-09	9.20E-10	4.43E-09	3.83E-09
ET2-bas	3.09E-08	2.67E-08	5.50E-09	4.75E-09	2.27E-08	1.96E-08
LN-ET	2.42E-15	2.81E-15	4.59E-15	3.82E-15	3.12E-15	3.13E-15
Bronch-bas	4.84E-09	4.34E-09	1.36E-09	1.22E-09	1.31E-09	1.18E-09
Bronch-sec	2.03E-08	1.82E-08	5.68E-09	5.11E-09	5.49E-09	4.94E-09
Bchiol-sec	1.41E-08	1.36E-08	2.19E-08	2.11E-08	7.18E-09	6.91E-09
AI	8.98E-12	7.34E-12	9.93E-10	8.12E-10	4.82E-10	3.94E-10
LN-Th	2.96E-15	1.91E-15	5.11E-15	3.79E-15	3.37E-15	2.44E-15
R-marrow	9.31E-14	7.37E-14	1.97E-13	1.56E-13	1.26E-13	1.00E-13
Endost-BS	4.57E-14	3.62E-14	9.67E-14	7.66E-14	6.21E-14	4.92E-14
Brain	4.09E-14	3.51E-14	8.67E-14	7.43E-14	5.56E-14	4.77E-14
Eye-lens	2.38E-14	2.00E-14	5.04E-14	4.22E-14	3.23E-14	2.71E-14
P-gland	3.79E-14	1.82E-14	8.01E-14	3.53E-14	5.14E-14	2.37E-14
Tongue	3.82E-14	5.43E-14	8.02E-14	1.14E-13	5.17E-14	7.35E-14
Tonsils	3.79E-14	1.66E-14	8.01E-14	3.50E-14	5.14E-14	2.25E-14
S-glands	3.78E-14	3.52E-14	8.01E-14	7.46E-14	5.14E-14	4.79E-14
Thyroid	8.33E-14	6.92E-14	1.76E-13	1.46E-13	1.13E-13	9.39E-14
Breast	1.52E-14	3.53E-14	3.21E-14	7.46E-14	2.06E-14	4.79E-14
Thymus	3.82E-14	3.55E-14	8.08E-14	7.50E-14	5.18E-14	4.81E-14
Ht-wall	9.08E-14	6.97E-14	1.92E-13	1.48E-13	1.23E-13	9.47E-14
Adrenals	9.86E-14	8.61E-14	2.09E-13	1.82E-13	1.34E-13	1.17E-13
Liver	1.12E-13	8.58E-14	2.37E-13	1.82E-13	1.52E-13	1.17E-13
Pancreas	1.04E-13	8.61E-14	2.20E-13	1.82E-13	1.41E-13	1.17E-13
Kidneys	1.44E-13	1.22E-13	3.05E-13	2.57E-13	1.95E-13	1.65E-13
Spleen	1.68E-13	1.37E-13	3.55E-13	2.91E-13	2.28E-13	1.87E-13
GB-wall	3.80E-14	3.54E-14	8.04E-14	7.49E-14	5.16E-14	4.81E-14
Ureters	3.78E-14	1.66E-14	8.01E-14	3.50E-14	5.14E-14	2.25E-14
UB-wall	3.58E-14	2.95E-14	7.57E-14	6.24E-14	4.86E-14	4.00E-14
Ovaries	8.62E-14	0.00E+00	1.83E-13	0.00E+00	1.17E-13	0.00E+00
Testes	0.00E+00	4.05E-14	0.00E+00	8.57E-14	0.00E+00	5.50E-14
Prostate	0.00E+00	3.53E-14	0.00E+00	7.46E-14	0.00E+00	4.79E-14
Uterus	1.52E-14	0.00E+00	3.21E-14	0.00E+00	2.06E-14	0.00E+00
LN-Sys	4.16E-14	3.39E-14	8.80E-14	7.17E-14	5.65E-14	4.60E-14
Skin	4.77E-14	3.66E-14	1.01E-13	7.73E-14	6.47E-14	4.96E-14
Adipose	3.15E-14	2.55E-14	6.64E-14	5.39E-14	4.27E-14	3.46E-14
Muscle	3.51E-14	2.90E-14	7.43E-14	6.13E-14	4.77E-14	3.93E-14

O-mucosa = oral mucosa, St-stem = stomach, RC-stem = right colon, RC-stem = rectosigmoid colon, ET2-bas = ET2 basal cells, Bronch-bas = bronchi basal cells, Bchiol-sec = bronchiolar secretory cells, LN-Sys = lymph nodes thoracic, Endost-BS = endosteal cells, Eye-lens = lens of eye, RS-stem = rectosigmoid colon, S-glands = salivary glands, Ht-wall = heart wall, GB-wall = gall bladder, UB-wall = urinary bladder.
